# Scavenger Receptor Mediates Systemic RNA Interference in Ticks

**DOI:** 10.1371/journal.pone.0028407

**Published:** 2011-12-01

**Authors:** Kyaw Min Aung, Damdinsuren Boldbaatar, Rika Umemiya-Shirafuji, Min Liao, Xuan Xuenan, Hiroshi Suzuki, Remil Linggatong Galay, Tetsuya Tanaka, Kozo Fujisaki

**Affiliations:** 1 Department of Pathological and Preventive Veterinary Science, The United Graduate School of Veterinary Science, Yamaguchi University, Yoshida, Yamaguchi, Japan; 2 Laboratory of Emerging Infectious Diseases, Department of Frontier Veterinary Medicine, Faculty of Agriculture, Kagoshima University, Korimoto, Kagoshima, Japan; 3 National Research Center for Protozoan Diseases, Obihiro University of Agriculture and Veterinary Medicine, Inada, Obihiro, Japan; Universidade Federal do Rio de Janeiro, Brazil

## Abstract

RNA interference is an efficient method to silence gene and protein expressions. Here, the class B scavenger receptor CD36 (*SRB*) mediated the uptake of exogenous dsRNAs in the induction of the RNAi responses in ticks. Unfed female *Haemaphysalis longicornis* ticks were injected with a single or a combination of *H. longicornis SRB* (*HlSRB*) dsRNA, vitellogenin-1 (*HlVg-1*) dsRNA, and vitellogenin receptor (*HlVgR*) dsRNA. We found that specific and systemic silencing of the *HlSRB*, *HlVg-1*, and *HlVgR* genes was achieved in ticks injected with a single dsRNA of *HlSRB*, *HlVg-1*, and *HlVgR*. In ticks injected first with *HlVg-1* or *HlVgR* dsRNA followed 96 hours later with *HlSRB* dsRNA (*HlVg-1/HlSRB* or *HlVgR/HlSRB*), gene silencing of *HlSRB* was achieved in addition to first knockdown in *HlVg-1* or *HlVgR*, and prominent phenotypic changes were observed in engorgement, mortality, and hatchability, indicating that a systemic and specific double knockdown of target genes had been simultaneously attained in these ticks. However, in ticks injected with *HlSRB* dsRNA followed 96 hours later with *HlVg-1* or *HlVgR* dsRNAs, silencing of *HlSRB* was achieved, but no subsequent knockdown in *HlVgR* or *HlVg-1* was observed. The Westernblot and immunohistochemical examinations revealed that the endogenous HlSRB protein was fully abolished in midguts of ticks injected with *HlSRB/HlVg-1* dsRNAs but HlVg-1 was normally expressed in midguts, suggesting that *HlVg-1* dsRNA-mediated RNAi was fully inhibited by the first knockdown of *HlSRB*. Similarly, the abolished localization of HlSRB protein was recognized in ovaries of ticks injected with *HlSRB/HlVgR*, while normal localization of HlVgR was observed in ovaries, suggesting that the failure to knock-down *HlVgR* could be attributed to the first knockdown of *HlSRB*. In summary, we demonstrated for the first time that SRB may not only mediate the effective knock-down of gene expression by RNAi but also play essential roles for systemic RNAi of ticks.

## Introduction

Ticks are obligate hematophagous ectoparasites of wild and domestic animals and humans. They are considered to be second to mosquitoes as vectors of human diseases and are the most important arthropods transmitting pathogens to domestic animals [1]. Double-stranded RNA (dsRNA)-mediated gene silencing, commonly referred to as RNA interference (RNAi), has been extensively used for the analysis of gene functions in ticks [2]. Long dsRNAs have been successfully and regularly applied in *Haemaphysalis longicornis* [3] and other tick species (e.g., *Amblyomma*, *Ixodes*, *Rhipicephalus*, and *Dermacentor* spp.) for targeted gene knockdown in various stages of tick life, with evidence of systemic RNAi spread into subsequent stages [2], [4]. Four different methods have been used to deliver dsRNA for RNAi in ticks to date: injection, soaking, feeding, and virus production of dsRNA [2]. We have confirmed that RNAi can be a powerful tool for gene silencing of the hard tick, *H. longicornis*, by the injection [3], [5]–[8] and soaking methods [9].

Direct injection of the dsRNA into target tissues or developmental stages is the most common method of delivering dsRNA to arthropods, such as insects and ticks [2], [10]. Injection of the exogenous dsRNAs into the insect’s hemocoel can provide transient knockdown of the target endogenous genes, since the dsRNA in the hemolymph can circulate systemically through the open circulatory system, in which dsRNA is taken up by a cell from the environment [11]. It is known that there are at least two pathways for exogenous dsRNA uptake in insects [12]. One is based on the transmembrane SID-1 channel protein, as well known from the *Caenorhabditis elegans* nematode. The second possible mechanism is based on the endocytosis-mediated pathway because it shares several components of its machinery with the dsRNA uptake mechanism. Herein, vacuolar H^+^ATPase is considered to play an important role [13]. However, the participation of scavenger receptors (SRs) already known to play a key role in microbe phagocytosis as “pattern recognition receptors” [14] is not well-established in dsRNA uptake.

SRs are known to potentially act as receptors for dsRNA molecules in an endocytosis-mediated uptake mechanism in the *Tribolium castaneum* beetle [15] and *Drosophila melanogaster* fly [13]. However, the involvement of SRs in dsRNA uptake and processing in the gene silencing of arthropods, including ticks, are not understood.

In a previous study, the gene encoding putative class B scavenger receptor (designated as *HlSRB*) was identified and characterized from *H. longicornis* [16]. The HlSRB had overall 30% identity to both mammalian and insect SRB membrane proteins. The mRNA transcripts of *HlSRB* were expressed in multiple organs of adult females but with varying levels in the different developmental stages of ticks. The recombinant HlSRB was expressed in *Escherichia coli* as the His-tagged protein, and anti-mouse recombinant HlSRB serum elucidated the localization of the endogenous protein in the midgut, salivary gland, ovary, fat body, and hemocytes of partially fed *H. longicornis* females. Gene silencing of *HlSRB* in female ticks resulted in a significant reduction of engorged body weights [16].

In this study, we elucidated the crucial role of *HlSRB* induction of knock-down of other endogenous genes via microinjections of a different combination of dsRNAs into the hemocoel of female ticks. RNAi has been proposed to have application possibilities for the autocidal control of tick populations [17] and the characterization of tick-borne pathogens [18], [4]. Therefore, a better understanding of the dsRNA uptake mechanism in tick RNAi will provide a comprehensive contribution to studies linked with the development of control measures for ticks and tick-borne diseases.

## Materials and Methods

### Ticks and animals

The parthenogenetic Okayama strain of the hard tick *H. longicornis* has been maintained by feeding on Japanese white rabbits (Kyudo, Kumamoto, Japan) in our laboratory [19]. Rabbit care was approved by the Animal Care and Use Committee of Kagoshima University (Approval no. A08010).

### Construction of dsRNA and microinjection of dsRNA into adult ticks

The dsRNA construction of *H. longicornis HlSRB* [16], *H. longicornis HlVgR* [6], and *H. longicornis HlVg-1* [7] and firefly *luciferase* (*luc*) as a control [16] was performed as described previously. The dsRNAs were injected into the hemocoel of unfed female ticks as described by Aung et al. [16]. The *HlSRB-*, *HlVgR-*, *HlVg-1-*, and *luc* dsRNA-injected ticks were allowed to rest at 25°C and 90% humidity regulated in an incubator for 96 hours to complete knock-down of these genes [16], [6], [7], and the mortality rate was then checked every 12 hours. Ninety-six hours after the first injection, three ticks were collected from the incubator in order to confirm gene-specific silencing by RT-PCR [16], [6], [7]. The remaining dsRNA-treated ticks were subjected to a second injection of dsRNAs.

Twelve tick groups injected with a single dsRNA or a combination of dsRNA(s) are as shown in Table 1. Each tick received a total of 0.5 µl dsRNA with a different concentration (for single dsRNA-injected groups, 1 µg/tick; for a combination of dsRNA(s)-injected groups, 1 µg/gene for a dose equal to the injected dsRNA at 2 µg/tick). The ticks injected with these dsRNAs were infested on the ear of rabbits 12 hours after the first or the second dsRNA injection. Four days after infestation, a total of 16 attached ticks were removed and collected from rabbits for the subsequent experiments including four ticks for RNA extraction, four ticks for protein lysate preparation, and eight ticks for tissue collection. The remaining ticks were allowed to feed until engorgement. To assess the effects of RNAi in ticks after the first and the second injections, we measured the number of ticks attached on a rabbit 2 days after attachment, the engorged body weight of ticks 5–6 days after attachment, the mortality rates, fecundities, and oocyte development of engorged ticks 20 days after engorgement, and the hatching rate to larvae 60 days after the first dsRNA injections.

**Table:** **Table 1.** Female tick groups injected with a single and a combination of dsRNA(s).

Tick groups	dsRNA used for the first injection	dsRNA used for the second injection
*HlSRB*	*HlSRB*	−
*HlVgR*	*HlVgR*	−
*HlVg-1*	*HlVg-1*	−
*HlSRB/HlVgR*	*HlSRB*	*HlVgR*
*HlSRB/HlVg-1*	*HlSRB*	*HlVg-1*
*HlSRB/luc*	*HlSRB*	*luc*
*HlVg-1/HlVgR*	*HlVg-1*	*HlVgR*
*HlVgR/HlSRB*	*HlVgR*	*HlSRB*
*HlVg-1/HlSRB*	*HlVg-1*	*HlSRB*
*luc/HlSRB*	*luc*	*HlSRB*
*HlVgR/HlVg-1*	*HlVgR*	*HlVg-1*
*luc*	*luc*	−

The first and second dsRNA injections were carried out at 96-hours interval.

### Collection of different tissues of dsRNA-injected ticks

Partially engorged female ticks 4 days post-infestation were removed from rabbits and dissected out for the subsequent tissue collection [19]. Midguts were collected from ticks injected with a single *HlSRB* or *HlVg-1* dsRNA and a combination of *HlSRB/HlVg-1*, *HlVg-1/HlVgR*, *HlVg-1,HlSRB*, or *HlVgR/HlVg-1* dsRNAs, ovaries were collected from ticks injected with a single *HlSRB* or *HlVgR* dsRNA and a combination of *HlSRB/HlVgR*, *HlVg-1/HlVgR*, *HlVgR/HlSRB*, or *HlVgR/HlVg-1* dsRNAs, and salivary glands and the fat bodies were collected from ticks injected with a single *HlSRB* or *luc* dsRNA and a combination of *HlSRB/luc* or *luc/HlSRB* dsRNAs.

### Reverse transcriptase-polymerase chain reaction (RT-PCR)

Whole bodies and dissected tissues from female ticks of each dsRNA-injected group fed for 4 days were subjected to total RNA extraction using a TRIzol reagent (Invitrogen, CA, USA). The RT-PCR analysis was performed using a one-step RNA PCR kit (Takara, Otsu, Japan) with the primer sets of *HlSRB* [16], *HlVgR* [6], and *HlVg-1* [7] genes. For all experiments, control amplification was carried out using the *H. longicornis β-actin-*specific primers (accession no. AY254898). The PCR products were subjected to electrophoresis in a 1.5% agarose gel in a TAE buffer; the DNA was visualized by ethidium bromide staining and analyzed using Quality One 1-D Analysis Software (Quantity One Version 4.5, Bio-Rad Laboratories, Milan, Italy).

### Protein expression analysis by Western blotting

The tick proteins from the lysates of whole bodies and the dissected tissues (about 1000 ng/lane) from female ticks of each dsRNA-injected group fed for 4 days were separated by 5-12% SDS-polyacrylamide gel electrophoresis and transferred to a polyvinylidene difluoride membrane (Millipore, MA, USA). The membrane was blocked with 5% skim milk in PBS-T (137 mM NaCl, 2.7 mM KCl, 10 mM Na_2_HPO_4_, 1.8 mM KH_2_PO_4_, 0.05% Tween-20, pH 7.4) and then incubated with 1∶100 dilution of anti-recombinant HlSRB (rHlSRB) [16], 1∶250 dilution of anti-rHlVgR [6], and 1∶200 dilution of anti-rHlVg-1 mouse sera [7] or anti-actin serum [8] as a first antibody. After the incubation of peroxidase-conjugated sheep anti-mouse IgG as a second antibody (1∶2000 dilution; GE Healthcare, Little Chalfont, UK), the specific protein bands were detected using 0.5 mg/ml 3,3'-diaminobenzidine tetrahydrochloride.

### Indirect immunofluorescent antibody test (IFAT)

The midguts, ovaries, salivary glands, and fat bodies dissected out from female ticks of each dsRNA-injected group fed 4 days were separately fixed with 4% paraformaldehyde in PBS including 0.1% glutaraldehyde at 4°C overnight. After washing with a sucrose series in PBS overnight, samples were embedded in Tissue-Tek O.C.T. Compound (Sakura Finetek, CA, USA) and frozen at −80°C. Frozen sections (12 µm) were cut with a cryostat (Leica CM 1850; Leica Microsystems, Wetzlar, Germany) and placed on micro-glass slides and then blocked with 5% skim milk in PBS overnight at 4°C. Sections were incubated for 30 minutes at 37°C with a 1∶100 dilution of an anti-rHlSRB, a 1∶200 dilution of anti-rHlVg-1, and a 1∶250 dilution of anti-rHlVgR mouse sera. After washing three times with PBS, Alexa 488- or Alexa 594-conjugated goat anti-mouse immunoglobulin (1∶1000; Invitrogen) was applied as a second antibody at 37°C for 1 hour. After washing three times with PBS, samples were mounted in a mounting medium with DAPI or Propidium Iodide (Vectashield; Vector Laboratories, Burlingame, CA, USA) and then covered with a cover glass. The images were photographed and recorded using a fluorescence microscope (Olympus, Tokyo, Japan).

### Statistical analyses

All statistical analyses were done with the Student’s *t*-test. Results are presented as the means ±SD. *P*<0.05 values were considered significant.

## Results

### dsRNA-mediated gene silencing of *HlSRB*, *HlVg-1*, and *HlVgR*

**Figure 1:**
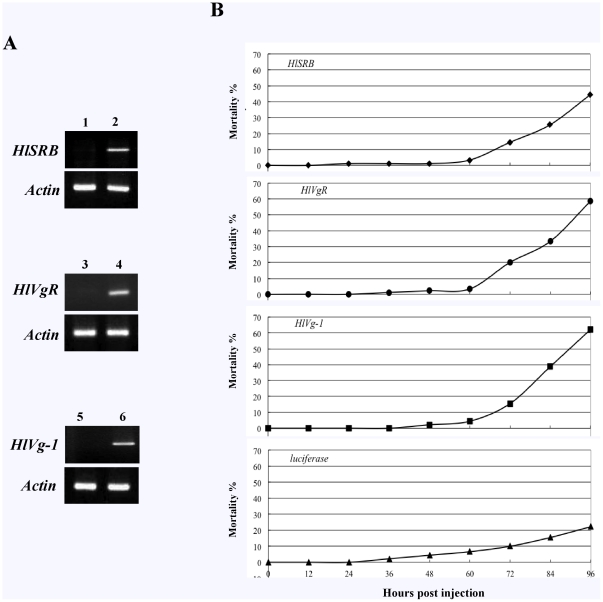
Gene silencing and mortality rate of *H. longicornis* 96 hours after a single dsRNA injection. dsRNA complementary to *HlSRB*, *HlVgR*, and *HlVg-1* was injected into *H. longicornis* adult females. The injected ticks were allowed to rest at 25°C in an incubator for four days to check mortality rates and gene silencing. RT-PCR analysis (A). PCR was performed using cDNA synthesized from three ticks injected with *HlSRB*, *HlVgR*, *HlVg-1*, or *luc* dsRNA with primer sets specific to *HlSRB*, *HlVgR*, *HlVg-1*, and the *β-actin* gene. Lane 1, *HlSRB* dsRNA-injected ticks; lanes 2, 4, and 6, *luc* dsRNA-injected ticks; lane 3, *HlVgR* dsRNA-injected ticks; lane 5, *HlVg-1* dsRNA-injected ticks. Mortality rates (B). Each panel represents treatment with one gene-specific dsRNA. Mortality rates were calculated by the percentage of number of dead ticks to the number of ticks used at the beginning of experiment in a different time course. *HlSRB*, *HlSRB* dsRNA-injected ticks; *HlVgR*, *HlVgR* dsRNA-injected ticks; *HlVg-1*, *HlVg-1* dsRNA-injected ticks; *luciferase*, *luciferase* dsRNA-injected ticks.

To investigate whether *HlSRB* knockdown has an effect on RNAi of other endogenous genes in ticks, we selected the *HlVg-1* and *HlVgR* genes. *HlVg-1* was transcribed only in the midgut [7], and the *HlVgR* gene, only in the ovary [6]. It was previously demonstrated that HlVg-1, one of the tick multiple vitellogenins (Vgs), is a crucial yolk protein precursor for oocyte development in ticks [7] and that HlVgR, a receptor localized on the surface of oocytes, plays a critical role for the specific binding with Vgs and the resultant Vgs transfer from hemolymph into oocytes via receptor-mediated endocytosis [6]. In this study, unfed female ticks were injected with a single *HlSRB*, *HlVgR*, or *HlVg-1* dsRNA for experimental groups as well as *luc* dsRNA as a control (Table 1). Ninety-six hours after the first injection, we observed apparent knockdown of the *HlSRB*, *HlVgR*, and *HlVg-1* transcriptions in ticks after the injection of respective dsRNAs by RT-PCR (Fig. 1A).

The mortality of ticks after dsRNA injections was examined every 12 hours. Forty-eight hours after injection, a slightly mortality was found in ticks of all groups (Fig. 1B). Seventy-two hours after injection, the mortality rate of ticks injected with the *luc* dsRNA was 10.0%, while those of the *HlSRB-*, *HlVgR-*, and *HlVg-1* dsRNA-injected tick groups were significantly higher, 14.4%, 20.0%, and 15.5%, respectively. Ninety-six hours after injection, the mortality rate of the *luc* dsRNA-injected ticks was 22.2%, while those of the *HlSRB-*, *HlVgR-*, and *HlVg-1* dsRNA-injected groups were significantly higher, 44.4%, 58.5%, and 62.2%, respectively. Four days after the first dsRNA injections, the live ticks were injected with second dsRNAs.

### Gene silencing of HlSRB, HlVgR, and HlVg-1 in whole bodies of female ticks injected with a single dsRNA or a combination of dsRNA(s)

**Figure 2:**
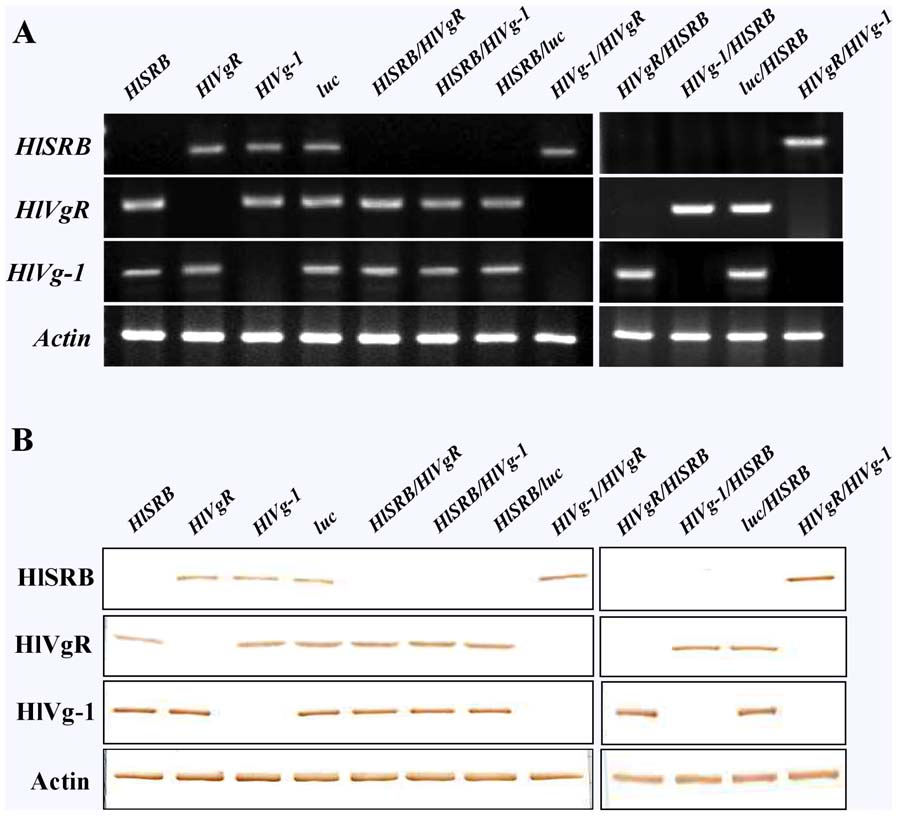
Silencing of HlSRB, HlVgR, and HlVg-1 genes and proteins in the whole body of *H. longicornis.* Individually or in combination of *HlSRB*, *HlVgR*, *HlVg-1*, and *luc* dsRNA(s) were injected into *H. longicornis* adult ticks. The injected ticks were left for 12 hours at 25°C and infested on the rabbits for four days and then ticks samples were collected for RNA extraction and the preparation of ticks protein lysates in each group. The name of each dsRNA group is indicated above. RT-PCR analysis (A). RT-PCR analysis was performed as shown in Fig. 1. (A). Western blot analysis (B). Tick lysates were subjected to SDS-PAGE under reducing conditions and transferred to a PVDF membrane. The membrane was probed with mouse anti-rHlSRB, anti-rHlVgR, or anti-rHlVg-1 sera; mouse anti-actin serum was used as a control. The name of each dsRNA group is the same as that used in Fig. 1.

RT-PCR and Western blot analysis were performed to elucidate the gene transcription and protein translation of HlSRB, HlVgR, and HlVg-1 in whole bodies of female ticks injected with a single dsRNA or a combination of dsRNA(s) (Table 1). As shown in Fig. 2A and B, the β-actin gene and protein levels did not change in all dsRNA-injected groups. A clear mRNA and protein knock-down of HlSRB, HlVgR, or HlVg-1 was observed in ticks injected with a single dsRNA of *HlSRB*, *HlVgR*, or *HlVg-1* (Fig. 2A and B). In ticks injected with a combination of *HlSRB/HlVgR*, *HlSRB/HlVg-1*, *HlSRB/luc*, *HlVgR/HlSRB*, *HlVg-1/HlSRB*, or *luc/HlSRB* dsRNAs, apparent mRNA and protein knock-down of HlSRB was attained in all groups regardless of whether *HlSRB* dsRNA was used for the first or second injection (Fig. 2A and B). In ticks injected with a combination of *HlVg-1/HlVgR*, or *HlVgR/HlVg-1* dsRNAs, clear double knockdown of endogenous *HlVg-1* and *HlVgR* genes was observed in both groups (Fig. 2A and B). Similar double gene knockdown was detected in ticks injected with a combination of *HlVgR/HlSRB* or *HlVg-1/HlSRB* dsRNAs (Fig. 2A and B). However, in ticks injected with *HlSRB* dsRNA followed 96 hours later with *HlVg-1* or *HlVgR* dsRNAs, no knockdown of *HlVgR* and *HlVg-1* was achieved, although clear gene silencing of *HlSRB* was attained, as described above (Fig. 2A and B), suggesting that RNAi mediated by the second *HlVgR* or *HlVg-1* dsRNA injection was inhibited by the first gene knockdown of *HlSRB*.

### mRNA and protein knock-down of HlSRB, HlVg-1, and HlVgR in different tissues of female ticks injected with a single dsRNA or a combination of dsRNA(s)

**Figure 3:**
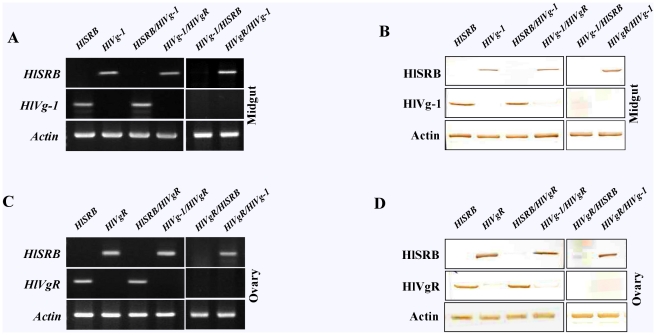
Expression profiles of HlSRB, HlVg-1, and HlVgR genes and proteins in different tissues of *H. longicornis*. Individually or in combination of *HlSRB*, *HlVg-1*, *HlVgR*, and *luc* dsRNA(s) were injected into *H. longicornis* adult ticks. The midguts and ovaries of dsRNA-injected ticks at 4 days of feeding were dissected out in 0.1% diethylpyrocarbonate-treated 1 × PBS (-) under a microscope. The name of each dsRNA group is indicated above. RT-PCR analysis and Western blot analysis were conducted using the midguts (A and B) and ovaries (C and D).

RT-PCR and Western blot analysis were performed to elucidate whether the mRNA and protein level in major internal organs, such as midguts, ovaries, salivary glands, and fat bodies, of female ticks attained single or double gene knockdown after various dsRNA injections (Fig. 3). As a result, mRNA and protein knock-down of HlSRB, HlVg-1, and HlVgR in the individual organs showed the same pattern of single or double gene knockdown as that observed using the whole bodies of female ticks (Fig. 2).

In the midguts (Fig. 3A and B), apparent silencing of *HlSRB* was observed after dsRNA injections with *HlSRB*, *HlSRB/HlVg-1*, or *HlVg-1/HlSRB*. Similar evident knockdown of *HlVg-1* was detected after dsRNA injections with *HlVg-1*, *HlVg-1/HlVgR*, *HlVg-1/HlSRB*, or *HlVgR/HlVg-1*. Double gene knockdown with *HlVg-1/HlSRB* injection and the failure to knock-down *HlVg-1* with *HlSRB/HlVg-1* injection were also pronounced in the midguts.

In the ovaries (Fig. 3C and D), silencing of *HlSRB* was observed after dsRNA injections with *HlSRB*, *HlSRB/HlVgR*, or *HlVgR/HlSRB*. Knockdown of *HlVgR* was detected after dsRNA injections with *HlVgR*, *HlVg-1/HlVgR*, *HlVgR/HlSRB*, or *HlVgR/HlVg-1*. Double gene knockdown with *HlVgR/HlSRB* injection and the failure to knock-down *HlVgR* with *HlSRB/HlVgR* injection were demonstrated.

In the salivary glands and fat bodies, mRNA and protein knock-down of HlSRB was observed after dsRNA injections with *HlSRB*, *HlSRB/luc*, and *luc/HlSRB* (data not shown).

### Immunofluorescent staining of the midguts, ovaries, salivary glands, and fat bodies

**Figure 4:**
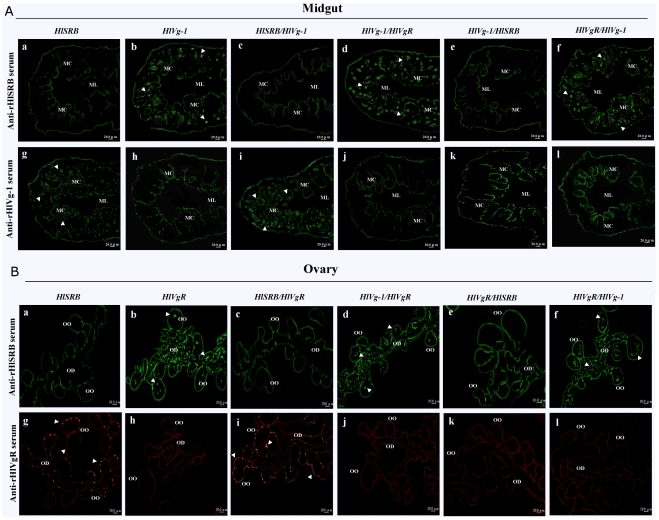
Confirmation of RNAi on the endogenous HlSRB, HlVg-1, and HlVgR in the different tissues of *H. longicornis*. The dissected tissues from the dsRNA-injected 4-days-feeding ticks were observed under fluorescence microscopy. The name of each dsRNA group is indicated above. The midguts were stained with anti-rHlSRB and anti-rHlVg-1 antibodies followed by Alexa 488-conjugated mouse anti-IgG with DAPI (A). Arrowheads indicate the native HlSRB and HlVg-1 expressed in the midguts. ML, midgut lumen; MC, midgut cells. The ovaries, staining pattern of anti-rHlSRB and anti-rHlVgR serum were used as first antibodies (B). The mouse anti-IgG conjugated with Alexa 488 was used as a second antibody with DAPI for the upper panels and Alexa 594 with Propidium Iodide for the lower panels. Arrowheads indicate the native HlSRB and HlVgR expressed in the ovaries. OO, oocyte; OD, oviduct. The *scale bar* represents 20 µm.

An immunohistochemical examination using an indirect fluorescent antibody test (IFAT) was conducted to illustrate the localization of the endogenous protein in the midguts, ovaries, salivary glands, and fat bodies of female ticks that exhibited single or double gene knockdown after the various dsRNA injections (Fig. 4).

As shown in Fig. 4A, no localization of HlSRB protein in midguts was observed after dsRNA injections with *HlSRB*, *HlSRB/HlVg-1*, or *HlVg-1/HlSRB* (panel a, c, and e). Similarly, no localization of HlVg-1 in midguts was observed after dsRNA injections with *HlVg-1*, *HlVg-1/HlVgR*, *HlVg-1/HlSRB*, or *HlVgR/HlVg-1* (panel h, j, k, and l). Neither HlVg-1 nor HlSRB protein localized in the midguts of ticks exhibited double gene knockdown by an injection of a combination of *HlVg-1/HlSRB* dsRNAs (panel e and k). Normal localization of the HlVg-1 protein in midguts, attributed to the failure to knock-down *HlVg-1* in *HlSRB/HlVg-1* injection, was also confirmed by IFAT (panel i).

In the ovaries, no localization of the native HlSRB protein was observed after dsRNA injections with *HlSRB*, *HlSRB/HlVgR*, or *HlVgR/HlSRB* (Fig. 4B, panel a, c, and e). No expression of HlVgR in ovaries was detected after dsRNA injections with *HlVgR*, *HlVg-1/HlVgR*, *HlVgR/HlSRB*, or *HlVgR/HlVg-1* (Fig. 4B, panel h, j, k, and l). Neither HlVgR nor HlSRB proteins localized in the ovaries of ticks exhibited double gene knock-down by an injection of a combination of *HlVgR/HlSRB* dsRNAs (panel e and k). The localization of HlVgR expressed in ovaries, thought to result from the failure to knock-down *HlVgR* with *HlSRB/HlVgR* injection, was also observed by IFAT (panel i).

In the salivary glands and fat bodies, no localization of native HlSRB protein was detected after dsRNA injections with *HlSRB*, *HlSRB/luc*, and *luc/HlSRB*, while HlSRB localization was observed after a single dsRNA injection of *luc* (data not shown).

### Engorgement, mortality and fecundity of female ticks injected with a single dsRNA or a combination of HlSRB, HlVgR, HlVg-1, and luc dsRNA(s)

Table 2 shows the phenotypic changes of adult female ticks injected with *HlSRB*, *HlVgR*, and *HlVg-1* dsRNA individually or in combination as well as *luc* dsRNA as a control (Table 1).

**Table:** **Table 2.** Phenotypic changes of ticks injected with a single or a combination of different dsRNA(s).

dsRNA groups	Number of ticks attached 24 h after infestation^a^	Average engorged body weight (mg)^b^	Mortality rate (%)^c^	Egg weight/body weight (%)^b^	Hatching rate (%)^d^
*HlSRB*	40	142.10±30.30^*^	7.5	41.95±11.25**	83.7
*HlVgR*	40	172.18±19.92^*^	5	11.62±12.41**	0
*HlVg-1*	40	81.56±18.16^*^	27.5	8.16±30.45**	13.7
*HlSRB/HlVgR*	38	143.61±14.38^*^	7.8	41.25±15.08**	82.8
*HlSRB/HlVg-1*	39	141.57±45.39^*^	7.6	42.10±16.32**	83.3
*HlSRB/luc*	39	142.81±17.12^*^	7.6	41.75±11.21**	83.3
*HlVg-1/HlVgR*	37	76.91±24.37^*^	86.4	0	0
*HlVgR/HlSRB*	38	150.29±11.32^*^	60.5	0	0
*HlVg-1/HlSRB*	38	85.81±64.14^*^	71	0	0
*luc/HlSRB*	38	143.15*±*32.63^*^	7.8	42.87±24.13**	82.8
*HlVgR/HlVg-1*	37	98.45±68.28^*^	91.8	0	0
*luc*	40	245.75±37.35	0	50.70±21.31	100

a Sixteen ticks were collected from the host for the subsequent experiments at 4 days after attachment.

b These ratios show the fecundity of engorged females. Values are the means of ±SD.

c These mortality rates show the percentages of number of dead ticks 20 days after drop-off to the total number of engorged ticks per treatment.

d Hatchings from eggs to larvae were examined at 25°C in an incubator for 60 days.

*P<0.05, luc dsRNA-injected group vs. HlSRB-, HlVgR-, HlVg-1-, HlSRB/HlVgR-, HlSRB/HlVg-1-, HlSRB/luc-, HlVg-1/HlVgR-, HlVgR/HlSRB-, HlVg-1/HlSRB-, luc/HlSRB-, and HlVgR/HlVg-1 dsRNA-injected groups.

**P<0.05, *luc* dsRNA-injected group vs. *HlSRB-*, *HlVgR-*, *HlVg-1-*, *HlSRB/HlVgR-*, *HlSRB/HlVg-1-*, *HlSRB/luc-*, and *luc/HlSRB* dsRNA-injected groups.

There were no differences in the groups in the number of ticks attached on a rabbit 24 hours after infestation (Table 2). The engorged body weight of ticks injected with a single dsRNA or a combination of *HlSRB*, *HlVgR*, *HlVg-1*, *HlSRB/HlVgR*, *HlSRB/HlVg-1*, *HlSRB/luc*, *HlVgR/HlSRB*, *luc/HlSRB* dsRNA(s) were significantly lower than those of ticks injected with a single *luc* dsRNA as a control (Table 2). It was evident that the engorged body weights of *HlVg-1* knockdowned ticks was conspicuously lower (Table 2).

The mortality rates 20 days after engorgement in ticks injected with a combination of *HlVg-1/HlVgR*, *HlVgR/HlSRB*, *HlVg-1/HlSRB* and *HlVgR/HlVg-1* dsRNAs, in which a double knockdown of targeted two genes was achieved (Fig. 2), were significantly higher, 86.4%, 60.5%, 71.0%, and 91.8%, respectively (Table 2). Most of the ticks from these groups died 18 hours after engorgement, and very few died within 5 days of engorgement. Mortality rates of 7.5%, 5.0%, and 27.5% were observed in ticks injected with a single dsRNA of *HlSRB*, *HlVgR*, and *HlVg-1* (Table 1). Similar lower mortality rates of 7.5%, 5.0%, 27.5%, 7.8%, 7.6%, 7.6%, and 7.8% were found in ticks injected with a single *HlSRB*, *HlVgR*, and *HlVg-1* dsRNA, respectively, or a combination of *HlSRB/HlVgR*, *HlSRB/HlVg-1*, *HlSRB/luc*, and *luc/HlSRB* dsRNAs, respectively (Table 2). These low mortality rates were observed in tick groups in which only a single knockdown of targeted genes was obtained (Fig. 2). No mortality was found in ticks injected with control *luc* dsRNA.

The fecundity of female ticks was estimated from the ratio of egg weight to engorged body weight. The ticks did not lay eggs in tick groups injected with a combination of *HlVg-1/HlVgR*, *HlVgR/HlSRB*, *HlVg-1/HlSRB*, and *HlVgR/HlVg-1* dsRNAs, in which double knockdown of targeted two genes was achieved, because the ticks could not achieve oviposition (Table 2). However, normal fecundity ratios were observed in tick groups with single gene knockdown, as described above (Table 2). The lower fecundity observed in ticks injected with a single *HlVg-1* dsRNA (Table 2) was attributed to the crucial involvement of Vgs in oocyte development in the ovaries.

The hatching rates from eggs to larvae were also examined at 25°C in an incubator for 60 days (Table 2). All eggs from ticks injected with a single dsRNA of *luc* succeeded in hatching to larvae (Table 2). In tick groups injected with a single dsRNA of *HlSRB* and a combination of *HlSRB/HlVgR*, *HlSRB/HlVg-1*, *HlSRB/luc*, and *luc/HlSRB* dsRNAs, in which single knockdown of *HlSRB* was achieved (Fig. 2), ca. 80% of hatching rates were observed (Table 2). The hatching rates of eggs laid by ticks showing single gene knockdown of *HlVgR* or *HlVg-1* after a respective dsRNA injection were evidently low, and eggs from the HlVgR dsRNA-injected ticks died without hatching (Table 2).

## Discussion

There are at least two pathways for exogenous dsRNA uptake in insects, such as the transmembrane channel protein-mediated and the endocytosis-mediated mechanisms [12]. In the latter endocytosis-mediated dsRNA uptake mechanism, the participation of SRs, known to be a key component of endocytosis [14], in dsRNA uptake was previously suggested in *T. castaneum* [15] and *D. melanogaster* insects [13]. However, it was unknown if SRs could have an important role in the dsRNA-mediated RNAi of ticks. To elucidate the role of SRs in dsRNA uptake in ticks, we used dsRNA of *HlSRB*, the class B scavenger receptor of *H. longicornis* ticks [16]. In the current study, dsRNA of *HlSRB* was injected into female ticks individually or in combination with different exogenous dsRNAs of ticks, namely *HlVg-1*, a yolk protein precursor expressed only at the midgut of *H. longicornis* [7], and *HlVgR*, a receptor localized only in the oocyte surface of *H. longicornis* [6], as well as firefly *luciferase* (*luc*) as a control [16].

As shown in Fig. 1A, the individual gene expression of the *HlSRB*, *HlVgR*, and *HlVg-1* was clearly inhibited in ticks 96 hours after a single dsRNA injection, indicating that mRNA knock-down had been successfully attained in *H. longicornis* ticks within 96 hours of the exogenous dsRNA injection. We found higher mortalities, from 44.4% to 62.2% in ticks 96 hours after a single dsRNA injection with *HlSRB*, *HlVgR*, or *HlVg-1* (Fig. 1B), while the mortality of control ticks injected with a *luc* dsRNA was 22.2%, which is not negligible. This result suggests that increased mortality in ticks injected with dsRNAs has been substantially attributed to the knockdown of targeted genes but partially associated with the possible external injuries, off-target effects associated with long dsRNA in RNAi screens, and functional impairments caused by microinjections in recipient ticks.

As shown in Fig. 2A and B, in the whole bodies of ticks injected with a combination of *HlVg-1/HlVgR* or *HlVgR/HlVg-1* dsRNAs, a clear and systemic double knockdown of endogenous *HlVg-1* and *HlVgR* genes was attained in both tick groups. Similar systemic double gene knockdown of targeted genes was observed in ticks injected with a combination of *HlVgR/HlSRB* or *HlVg-1/HlSRB* dsRNAs. However, in the whole bodies of ticks injected with a combination of *HlSRB/HlVgR* or *HlSRB/HlVg-1* dsRNAs, no knockdown of *HlVgR* and *HlVg-1* was achieved, although clear systemic gene silencing of *HlSRB* was attained. These results suggest that systemic RNAi mediated by the second *HlVgR* or *HlVg-1* dsRNA injection was dramatically inhibited by the first systemic gene knockdown of *HlSRB*. No conclusion can yet be drawn, but it is speculated that SID-1, SRs, vacuolar H^+^ATPase, RDS-3, and RdRp could be possible components of the dsRNA uptake mechanism in several insects [12]. With regard to SRs, two scavenger receptors of *D. melanogaster*, SR-CI and Eater, account for more than 90% of dsRNA uptake by S2 cells [13]. Our results clearly indicate that HlSRB, class B scavenger receptor of *H. longicornis* ticks is essential for systemic RNAi/effective knock-down of gene expression by RNAi.

Boldbaatar et al. [6], [7] demonstrated that the transcription and translation of HlVg-1 are midgut-specific and those of HlVgR are ovary oocyte-specific. In the current study, we examined the expression and localization of HlVg-1 in midguts and HlVgR in ovaries of female ticks showing a single or double systemic RNAi. The localization of HlSRB was also examined in midguts, ovaries, salivary glands, and fat bodies. As a result, normal expression of the gene and protein of HlVg-1 in midguts and HlVgR in ovaries were clearly shown in female ticks with a systemic RNAi of *HlSRB* (Figs. 3 and 4).

Systemic RNAi can only take place in multicellular organisms because it includes processes in which a silencing signal is transported from one cell to another or from one tissue type to another [12]. In multicellular organisms, such as ticks, the *HlVg-1* or *HlVgR* dsRNA internalized through injection into hemocoel must be taken up from the hemolymph to the midgut cells or ovary oocytes in order to silence the target genes. We conclude that the dysfunction of receptor-mediated endocytosis was introduced in ticks by the systemic RNAi of *HlSRB*, resulting in the uptake abrogation of *HlVg-1* or *HlVgR* dsRNA in midguts or ovaries and leading to normal protein expression.

Most of our understanding of tick RNAi is mediated systemic delivering of RNAi effect and the literature demonstrated that a systemic RNAi silencing mechanism is active in ticks [3]–[9]. Results obtained in this study might explain that the SR-mediated dsRNA uptake mechanism is evolutionarily conserved in ticks and plays a crucial role in controlling the induction of systemic gene silencing in ticks. However, other factors, such as proteins of the vesicle-mediated transport, conserved oligomeric Golgi complex family, cytoskeleton organization and protein transport are directly and/or indirectly involved in endocytosis and known to play roles in dsRNA uptake and processing [20]. Further studies are needed to examine these factors in *H. longicornis*. These results may contribute to the development of a control strategy for ticks and pathogen transmission.

The overall results presented in this study show that dsRNAs of *HlSRB*, *HlVg-1*, and *HlVgR* introduced into ticks individually or in combination resulted in different but significant phenotypic changes in them (Table 2). These phenotypes in engorgement, mortality, fecundity, and oocyte development of ticks were comparable with those in previous characterization [6], [7], [16] and were more prominent in ticks indicating double knockdown of *HlVg-1/HlVgR*, *HlVgR/HlSRB*, or *HlVg-1/HlSRB* genes than in those with single gene knockdown of *HlSRB*, *HlVg-1*, or *HlVgR* (Table 2), suggesting that systemic and specific double knockdown of target genes had been simultaneously attained in these ticks. This successful double gene knockdown might show promising application possibilities for combinational RNAi in the practical control measures of ticks and tick-borne diseases.

In summary, research in recent years has given new insights into the dsRNA uptake mechanism in the gene silencing of insects. However, the role of dsRNA uptake in ticks remains to be proven. We demonstrated for the first time in the current study, using *HlSRB*, a class B scavenger receptor CD36 of *H. longicornis*, that *SRB* mediates the effective knock-down of gene expression by RNAi and plays essential roles for the systemic RNAi of ticks.
